# Longitudinal assessment of oral intake adequacy and nutrition-impacted symptoms in patients undergoing allogeneic hematopoietic stem cell transplantation

**DOI:** 10.3389/fnut.2025.1646941

**Published:** 2025-12-03

**Authors:** Liyuan Qin, Rui Li, Jiaqi Wu, Yang He

**Affiliations:** Department of Nursing, Ruijin Hospital, Shanghai Jiao Tong University School of Medicine, Shanghai, China

**Keywords:** oral intake, adequacy, nutrition impacted symptoms, oral nutrition supplements, allogeneic hematopoietic stem cell transplantation

## Abstract

**Aims:**

This study aims to longitudinally assess the adequacy of oral intake and burden of nutrition-impacted symptoms (NIS) and identify modifiable factors associated with oral intake adequacy in allogeneic hematopoietic stem cell transplantation patients.

**Methods:**

A single-center prospective study enrolled allogeneic hematopoietic stem cell transplantation patients from May 19, 2022 to February 8, 2023. We evaluate the adequacy of oral intake and nutrition-impacted symptoms in the peritransplantation period. Generalized estimating equations were adopted to evaluate associated factors of the adequacy of oral energy and protein intake.

**Results:**

A total of 63 allogeneic hematopoietic stem cell transplantation patients were enrolled in the study, with 439 records. The adequacy of oral energy/protein intake showed a trend of decreasing first and then increasing, and 57.1–95.2% of the patients suffered from at least one nutritionally impacted symptom in the peritransplantation period. Generalized estimating equations indicated that the presence of nutrition impacted symptoms was a risk factor significantly associated with the adequacy of oral energy intake <50%, while the use of oral nutrition supplements was a protective factor. There were similar associations between the adequacy of oral protein intake <50% and the above two factors.

**Conclusion:**

Insufficient oral energy and protein intake and the burden of nutrition impacted symptoms in peritransplantation patients are common, and further attention and intervention on these issues are needed. Oral nutrition supplements are a potential approach to increase oral intake and nutritional status in allogeneic hematopoietic stem cell transplantation patients.

## Introduction

Allogeneic hematopoietic stem cell transplantation (allo-HSCT) has been an effective and widely used treatment for malignant cancers of the blood system (1). Allo-HSCT patients must receive multiple high-dose chemical and/or systemic radiation treatments before transplantation and often suffer from complications such as opportunistic infection and graft-versus-host disease after transplantation, which often leads to digestive dysfunctions that cause a high risk of developing malnutrition. A previous survey conducted in China showed that 1.76% of patients experienced mild to severe malnutrition before allo-HSCT, a number that increased to 83.3% after HSCT (2). Poor nutritional condition in allo-HSCT patients may lead to reduced tolerance to the conditioning regimen, delayed hematopoietic immune reconstitution, and the status before and after transplantation significantly impacts outcomes, including length of hospital stay and overall survival (3–5). Thus, timely and adequate nutritional support is in urgent need of attention in patients undergoing allo-HSCT.

The primary goal of nutritional support is to ensure adequate energy and protein intake, and allo-HSCT patients should have an energy intake of at least 25–30 kcal/day and a protein intake of at least 1.5–2 g/day according to the guidelines (6, 7). Oral nutrition is the preferred approach for nutritional support as it allows for the digestion and absorption of nutrients in line with the physiological state, which helps maintain the physiological functions of the digestive tract and intestinal mucosal barrier (4, 6). Most allo-HSCT patients can eat orally in the peritransportation (7). Encouraging patients to eat orally independently is the main approach of nutritional support and utilization of total parenteral nutrition only when patients are unable to consume at least 50% of their energy needs for ≥3 days (7). However, there are limited studies on analyzing the oral nutrition intake of allo-HSCT patients in the peritransplantation period, and the gap between the actual intake of energy, protein, and other key nutrients and the target requirements of patients remains unknown. Therefore, understanding the oral intake states and issues related to it in the allo-HSCT population is essential to optimize oral energy and protein intake throughout transplantation and initiate advanced nutrition support as soon as necessary. Nutrition-impacted symptom (NIS) is defined as a group of symptoms that reduce oral intake and lead to weight loss (8, 9). According to the ESPEN guideline, the treatment of symptoms and derangements impairing food intake (nutrition impact symptoms) is a vital part to increase oral intake in cancer patients (6). But previous studies on allo-HSCT patients have focus on distressing and physical symptoms or assessed only partial NIS (10) and lack reports on a comprehensive assessment of NIS.

Therefore, this study aims to: Determine the longitudinal trajectory of the adequacy of oral intake and burden of nutrition-impacted symptoms (NIS),and identify factors associated with inadequate oral intake in allo-HSCT patients.

## Methods

### Study population and design

In this longitudinal study, participants were recruited using a consecutive sampling approach. Individuals aged 18 or above who underwent allogeneic HSCT for a hematologic malignancy at Ruijin Hospital of Medical School of Shanghai Jiaotong University in China between May 2022 and February 2023 were eligible. Patients who suffered from an interruption or termination of transplantation for the change of disease condition, or had comorbidities affecting digestion and absorption and required parenteral nutrition (PN) support during transplantation were excluded. This study is approved (No. 127/RH-21) by the Ruijin Hospital Ethics Committee, Shanghai Jiao Tong University School of Medicine, and all participants involved in the study signed an informed consent.

### General information

Data on demographic and disease characteristics, is age, gender, residence, medical diagnosis, conditioning regimen, donor, and use of oral nutrition supplements (ONS) were obtained from the patient’s medical records. This was an observational study. Oral nutrition supplements were prescribed at the discretion of the medical team and not as part of a predefined intervention protocol. All the oral nutritional supplements applied to the research subjects were of standard types, with energy density and protein density being 450 kcal/100 g and 15.9 g/100 g, respectively.

### Oral intake assessment

In this study, dietary intake was assessed using the 24-h dietary record by an experienced trained nurse. The nurse recorded participants’ food before and after breakfast, lunch, and dinner, as well as extra meals such as ONS and snacks at seven time points, including the first day after admission, day 2 of the conditioning regimen, day 1, 7, 14, 21 after transplantation, and the day before discharge, at which oral intake varies greatly due to the treatment, as observed in the recent clinical practice. To ensure the accuracy of the assessment, a food model diagram was used to assist in assessing the dietary intake of patients. Records were analyzed using the Nutrition Therapeutic System of Traditional Chinese Medicine Combined with Western Medicine MX2 to obtain the actual intake of energy and protein.

According to the ESPEN guideline (5) and the guideline for patient care in blood and marrow transplant (7), the recommendation for the energy need of HSCT patients is 25–30 kcal/kg, while the protein need is 1.5–2 g/kg. Thus, in this study, the target energy need was calculated as weight *Χ* 25 kcal/kg, while the target protein need was calculated as weight X 1.5 kcal/kg. For participants with obesity (BMI ≥ 28 kg/m^2^), adjusted weight: [ideal weight + 0.025 (actual weight − ideal weight)] was used to replace weight (7). In particular, deal weight was calculated as height^2^ (m) × 22.5 (11). The respective adequacy of oral energy/protein intake was defined by comparing actual oral energy/protein intake with the target energy/protein needs. The adequacy rates ranged between 0 and 100%. If more than the target amount of energy/protein was consumed, the rate was truncated at 100%.

### Nutrition impacted symptoms

Nutrition impacted symptoms were assessed with the symptom section of the Patient-Generated Subjective Global Assessment (PG-SGA) (12), which includes 16 symptoms (no problem, no appetite, nausea, vomiting, diarrhea, oral mucositis, smells bother me, feel full quickly, constipation, things taste funny or no taste, dry mouth, problems swallowing, pain, other: depression, money, dental problems) related to decreased food intake. Patients were asked about the presence of the NIS (yes/no) in the past week.

### Statistical analysis

Data analysis was conducted using IBM SPSS 25.0. The categorical variables were expressed as numbers and proportions, and the continuous variables were shown in means and standard deviations (SDs) when meeting normal distribution or medians with interquartile range (IQR) when they are not normally distributed.

To take the within-subject correlation into account, generalized estimating equations (GEE) with an unstructured correlation were adopted to comprehensively evaluate the association between longitudinal adequacy of oral energy/protein intake and demographic as well as disease characteristics. Variables were entered into a GEE model separately for univariate analysis first, and then those with a *p* < 0.1 were included in the GEE model for multivariable analysis.

## Results

### Baseline characteristics of the participants

During May 2022 and February 2023, 70 patients were enrolled at baseline, of whom 63 were included for final analysis. An additional 4 patients were excluded due to an interruption of transplantation for complications, and 3 were excluded due to receiving TPN due to severe oral mucositis (*N* = 1)or acute intestinal graft-versus-host disease (*N* = 2). Sixty-one participants completed all 7 time points follow-up survey, while 2 were discharged earlier than 21 days after transplantation and missed the data in the sixth time point. The baseline characteristics of the participants are shown in Table 1. Most (*n* = 38, 60.3%) were diagnosed with acute myelogenous leukemia (AML) and 79.4% (*n* = 50) of them received a conditioning regimen of FBM and the source of hematopoietic stem cells from a haploidentical donor. Two of the participants accepted TPN due to gut graft-versus-host disease, while one of them accepted TPN because of severe oral mucositis during the survey.

**Table 1:** Baseline characteristics of the participants.

Variables	N/Median (Percentage/IQR)
Age (years old)	49 (37, 55)^a^
Gender	
Male	29 (46)
Female	34 (54)
Education level	
Elementary or below	8 (12.7)
Middle school	18 (28.6)
High school	12 (19)
College or above	25 (39.7)
Residence	
Rural area	28 (44.4)
Urban area	35 (55.6)
Percentage of weight loss during 21d after allo-HSCT (%)	9.91 (8.67, 12.15)
BMI at admission(kg/m^2^)	
<18.5	3 (4.8)
18.5–23.9	39 (61.9)
24–27.9	18 (28.6)
≥28	3 (4.8)
Disease diagnosis	
Acute myelogenous leukemia	38 (60.3)
Acute lymphocyte Leukemia	15 (23.8)
Other (granulocytic sarcoma or myelodysplasia syndrome)	10 (15.9)
Conditioning regimen	
FBM	50 (79.4)
VP16+FBM	9 (12.7)
FB	4 (6.3)
Donor	
Haploidentical donors	50 (79.4)
Matched related donors	8 (12.7)
Matched unrelated donors	5 (7.9)
The use of ONS	
Yes	25 (39.7)
No	38 (60.3)

^a^Data are expressed as median(IQR). IQR, interquartile range; FB, Flu+Bu; FBM, Flu+Bu+Mel; VP16+FBM, Flu+Bu+Mel+VP16; ONS, oral nutrition supplements; BMI, body mass index.

### Longitudinal adequacy of oral energy/protein intake

**Figure 1:**
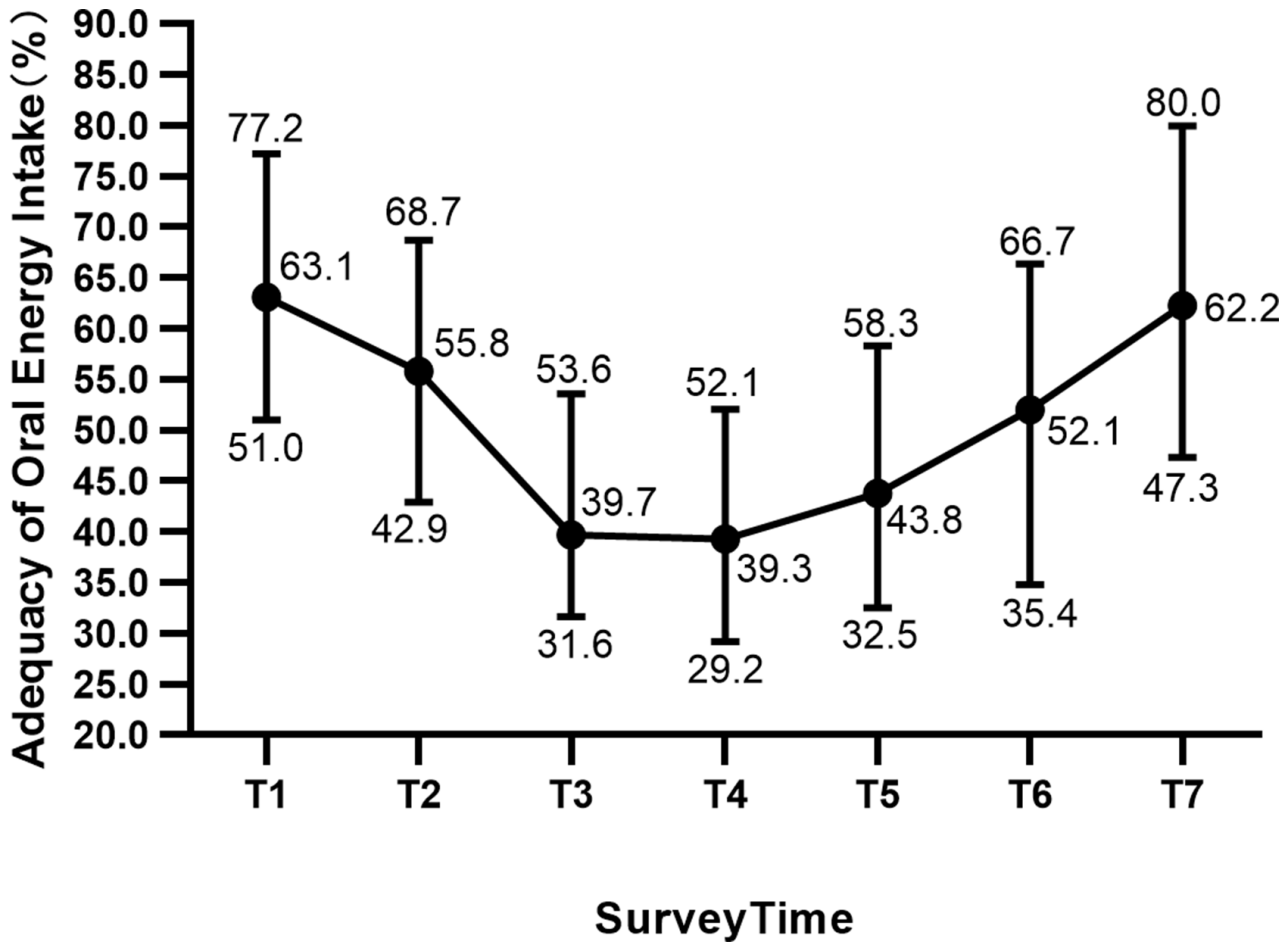
Adequacy of oral energy intake (%) in patients undergoing allogeneic hematopoietic stem cell transplantation. The median (dots) with 75% percentile (upper bars) and 25% percentile (lower bars) are shown in the figure. T1: the first day after admission, T2: day 2 of the conditioning regimen, T3–T6: day 1, 7, 14, 21 after transplantation; T7: the day before discharge.

**Figure 2:**
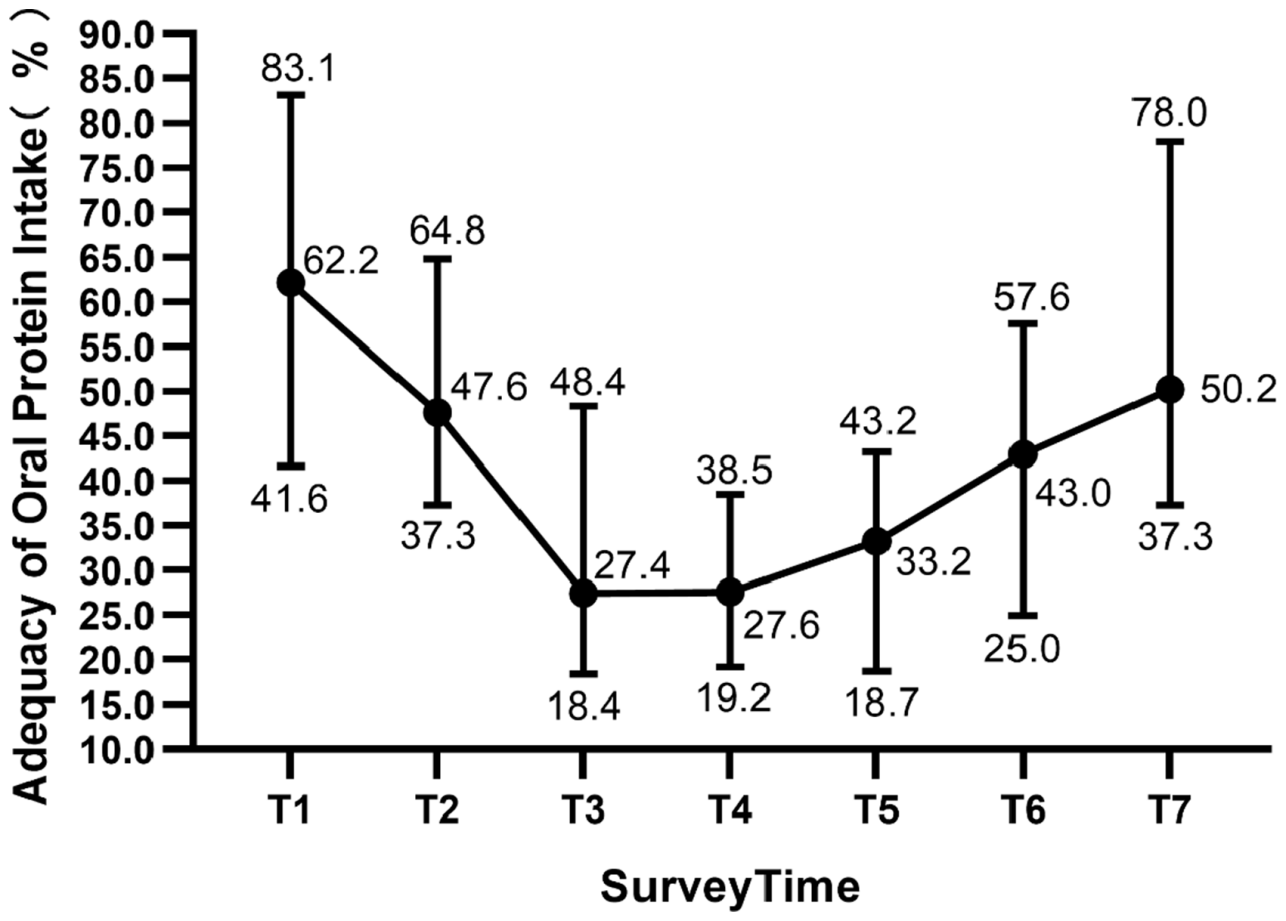
Adequacy of oral protein intake in patients undergoing allogeneic hematopoietic stem cell transplantation. The median (dots) with 75% percentile (upper bars) and 25% percentile (lower bars) are shown in the figure. T1: the first day after admission, T2: day 2 of the conditioning regimen, T3–T6: day 1, 7, 14, 21 after transplantation; T7: the day before discharge.

The median adequacy of oral energy intake in allo-HSCT patients declines from the day of admission (63.1%, IQR 51.0–77.2%) to the day 14 after transplantation (39.26%, IQR 42.9–68.7%), whereas it rises from the day 14 to the day of discharge (62.24%, IQR 47.3–80.0%) (Figure 1). Further analysis showed that the proportion of patients with oral energy intake <50% of the targeted intake was 22.2, 39.7, 68.3, 73.0, 55.6, 45.9, and 31.7% at the seven survey time points, respectively. A similar pattern was seen within the adequacy of oral protein intake in allo-HSCT patients, which decline from the day of admission (62.2%, IQR 41.6–83.1%) to the day 7 after transplantation (27.43%, IQR 18.4–48.4%), and then rise to the day of discharge (50.24%, IQR 37.3–78.0%) (Figure 2). The proportion of patients consuming <50% of the target protein intake was 31.7, 55.6, 77.8, 88.9, 84.1, 65.6, and 47.6% at the seven survey time points, respectively.

### Longitudinal nutrition impacted symptoms of the participants

**Figure 3:**
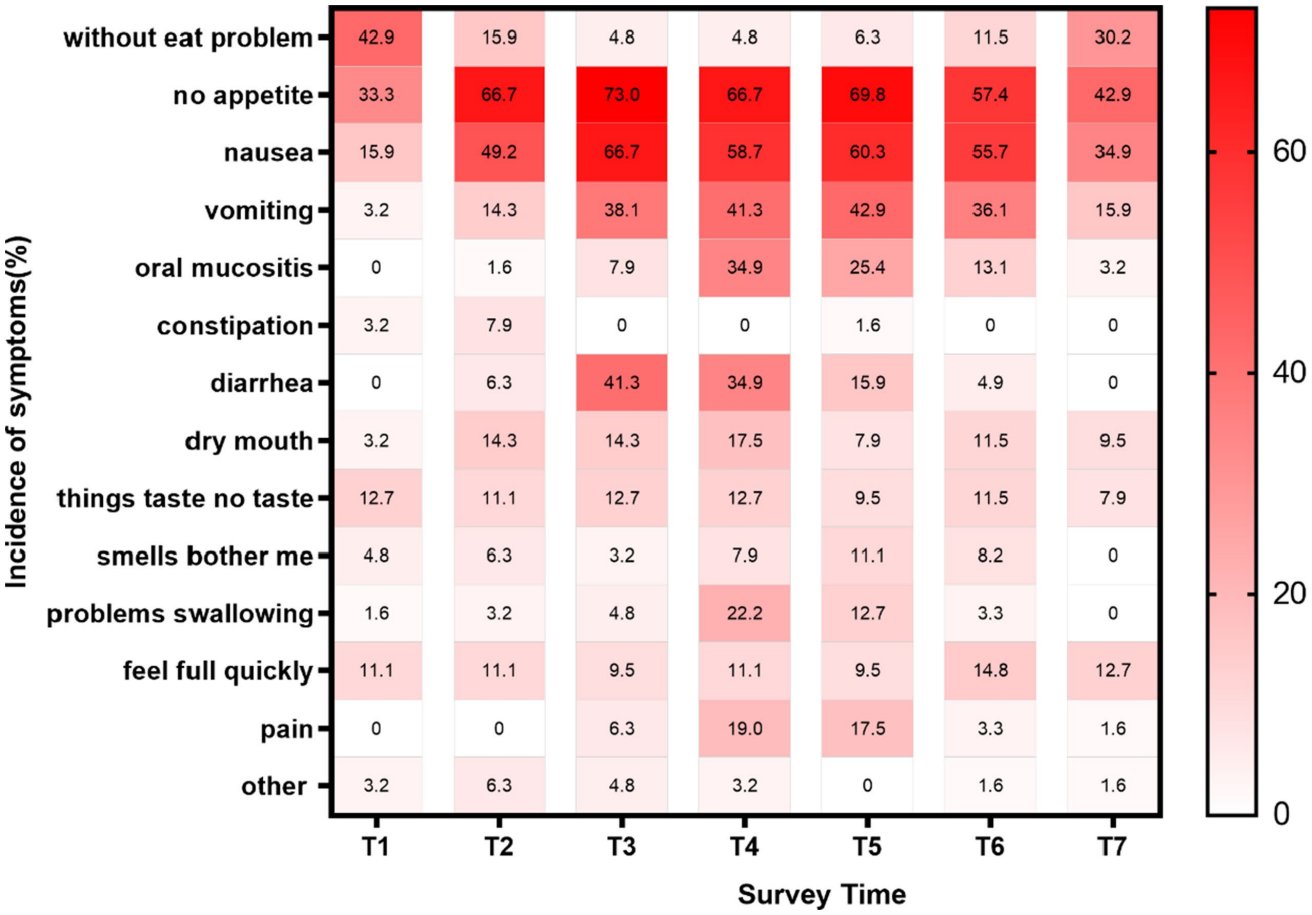
Heatmap of incidence of nutrition impacted symptoms during allogeneic hematopoietic stem cell transplantation. Other: depression, money, dental problems; T1: the first day after admission, T2: day 2 of the conditioning regimen, T3–T6: day 1, 7, 14, 21 after transplantation; T7: the day before discharge.

As is shown in Figure 3, more than half (57.1–95.2%) of patients suffered from at least one nutritionally impacted symptom in the peritransplantation period. And NIS is of the highest prevalence at day 1 and day 7 after transplantation, reported in 95.2% of patients (*n* = 63). No appetite is the most common NIS in the peritransplantation period, with prevalence ranging from 33.3 to 73%, followed by nausea (15.9–66.7%) and vomiting (3.2–42.9%).

### Factors associated with adequacy of oral energy intake

According to the result of univariate analysis, 4 variables (time point, conditioning regimen, the use of ONS, and the presence of NIS) were included for multivariable analysis (Table 2). Compared to the day of admission, a significantly higher risk of the adequacy of oral energy intake <50% was detected on day 2 of the conditioning regimen, day 1, 7, 14, 21 after transplantation, while no significance was found on the day before discharge (wald c^2^ = 1.559, *p* = 0.212). As to the conditioning regimen, allo-HSCT patients who accepted the conditioning regimen of the FBM regimen are at a higher risk of consuming <50% of the target energy compared to those with the conditioning regimen of Flu+Bu (FB) (OR = 1.920, *p* = 0.018). Patients using ONS in the peritranplantation period were 86.7% less likely with the adequacy of oral energy intake <50% (OR = 0.133, *P <* 0.001). And compared with those without the burden of NIS, allo-HSCT patients who suffered from ≥1 NIS were 135.9% more likely with the adequacy of oral energy intake <50% (OR = 2.359, *p* = 0.012).

**Table 2:** Generalized estimating equation analysis of factors associated with the adequacy of oral energy intake in allo-HSCT patients.

Variables	*β*	SE	OR (95%CI)	Wald c^2^	*p* value
Intercept	−1.801	0.398	0.165 (0.076, 0.360)	20.537	<0.001
Time point					
T7	−0.574	0.408	1.664 (0.748, 3.700)	1.559	0.212
T6	−1.400	0.418	3.275 (1.443, 7.433)	8.051	0.005
T5	−1.885	0.430	5.190 (2.235, 12.054)	14.671	<0.001
T4	−2.738	0.456	12.215 (5.012, 29.771)	30.314	<0.001
T3	−2.290	0.411	7.592 (3.394, 16.981)	24.356	<0.001
T2	−0.835	0.303	1.849 (1.021, 3.348)	4.117	0.042
T1	–	–	–	–	–
Conditioning regimen					
FBM	0.652	0.275	1.920 (1.120, 3.289)	5.634	0.018
VP16+FBM	−0.121	0.414	0.886 (0.393, 1.996)	0.085	0.770
FB	–	–	–	–	–
Use of ONS					
Yes	−2.018	0.387	0.133(0.062, 0.284)	27.228	<0.001
No	–	–	–	–	–
NIS					
NIS ≥ 1	0.858	0.343	2.359(1.206, 4.617)	6.278	0.012
NIS = 0	–	–	–	–	–

Parameter setting of the dependent variable in the Generalized estimating equation model: 1 = the adequacy of oral energy intake <50%; 0 = the adequacy of oral energy intake ≥50%. T1: the 1st day after admission, T2: day 2 of the conditioning regimen, T3–T6: day 1, 7, 14, 21 after transplantation; T7: the day before discharge. Allo-HSCT, allogeneic hematopoietic stem cell transplantation; SE, standard error; OR, odds ratio; CI, confidence interval; FB, Flu+Bu; FBM, Flu+Bu+Mel; VP16+FBM, Flu+Bu+Mel+VP16; ONS, oral nutrition supplements; NIS, nutrition impacted symptoms.

### Factors associated with adequacy of oral protein intake

As for the adequacy of oral protein intake, time point, gender, residence, the use of ONS during, and the presence of NIS were included in the GEE model for multivariable analysis. As is shown in Table 3, compared to the day of admission, significantly increased risks of the adequacy of oral protein intake <50% were reported on day 2 of the conditioning regimen, day 1, 7, 14, 21 after transplantation, while no significance was found on the day before discharge. There is no significance between patients of different genders and residences. Patients using ONS were 61.3% less likely to consume <50% of the target protein intake compared to those without the use of ONS (OR = 0.387, *p* = 0.001). And allo-HSCT patients who suffered from ≥1 NIS were almost 1 time more likely with the adequacy of oral energy intake <50% (OR = 2.007, *p* = 0.048) compared to patients who suffered no NIS.

**Table 3:** Generalized estimating equation analysis of factors associated with the adequacy of oral protein intake in allo-HSCT patients.

Variables	*β*	SE	OR (95%CI)	Wald c^2^	*p* value
Intercept	−0.670	0.382	0.512 (0.242, 1.082)	3.075	0.080
Time point					
T7	0.672	0.404	1.958 (0.887, 4.319)	2.768	0.096
T6	1.463	0.422	4.319 (1.890, 9.870)	12.038	0.001
T5	2.514	0.473	12.356 (4.983, 31.202)	28.298	<0.001
T4	2.923	0.523	18.599 (6.687, 51.729)	31.370	<0.001
T3	1.982	0.428	7.258 (3.139, 16.783)	21.479	<0.001
T2	0.860	0.332	2.364 (1.232, 4.534)	6.703	0.010
T1	–	–	–	–	–
Gender					
Female	−0.467	0.313	0.627 (0.340, 1.158)	2.222	0.136
Male	–	–	–	–	–
Residence					
Urban area	−0.514	0.308	0.598(0.327, 1.095)	2.772	0.096
Rural area	–	–	–	–	–
The use of ONS					
Yes	−0.949	0.276	0.387(0.225, 0.665)	11.821	0.001
No	–	–	–	–	–
NIS					
NIS ≥ 1	0.697	0.352	2.007(1.006, 4.003)	3.913	0.048
NIS = 0	–	–	–	–	–

Parameter setting of the dependent variable in the Generalized estimating equation model: 1 = the adequacy of oral protein intake <50%; 0 = the adequacy of oral protein intake ≥50%. T1: the first day after admission, T2: day 2 of the conditioning regimen, T3–T6: day 1, 7, 14, 21 after transplantation; T7: the day before discharge. Allo-HSCT, allogeneic hematopoietic stem cell transplantation; SE, standard error; OR, odds ratio; CI, confidence interval; FB, Flu+Bu; FBM, Flu+Bu+Mel; VP16+FBM, Flu+Bu+Mel+VP16; ONS, oral nutrition supplements; NIS, nutrition impacted symptoms.

## Discussion

This study provides a longitudinal evaluation of the adequacy of oral energy and protein intake in allo-HSCT patients, which indicates that peri-transplant oral intake of energy and protein is inadequate. Most of the allo-HSCT patients were suffering from various NIS in different periods of peritransplantation. These data provide strong evidence that oral nutrition supplements and specific management of symptoms should be conducted to eliminate the gap between oral intake and its target.

Allo-HSCT patients are in a hypercatabolic state with significantly increased demands for energy and protein related to wound healing after conditioning regimens, infectious events with associated febrile states, and the systemic inflammatory state and local tissue damage imposed by acute graft versus host disease (6). Maintaining adequate nutritional balance throughout the transplant process is critically important. However, due to disease and treatment-related digestive symptoms, lack of knowledge of nutrition, and inadequate health education, HSCT patients tend to have reduced dietary intake and poor dietary quality (13, 14). Previous survey on 10 allo-HSCT patients showed that the caloric and protein adequacy first decreased and then rose during hospitalization, with the caloric adequacy being 56.5–83.7% and the protein adequacy being 49.2–72.3% (15), a similar trend was shown in our recent survey in a larger sample of patients. Importantly, weight loss ≥10% was associated with during the 3 months after allogeneic HSCT has been strongly associated with adverse long-term outcomes, including increased non-relapse mortality and worse OS (16). Our survey found that the median percentage of weight loss during transplantation was 9.91%, and supplementary analysis revealed significant correlations between percentage weight loss and both energy intake adequacy and protein intake adequacy during transplantation. Regular assessment of dietary intake and timely adequate enteral nutrition support is associated with reduced non-relapse mortality, improved survival, and GvHD-free/relapse-free survival at 5 years in allo-HSCT patients (17). In comparison with the prior study, we further revealed the gaps between actual energy and protein intake and the target intake in seven different peritransplantation periods, which demonstrated that oral energy and protein intake in most allo-HSCT patients are inadequate, and the necessity of nutritional follow-up during hospitalization to meet the nutritional needs should be emphasized.

ONS refers to supplementary oral intake of dietary food for special medical purposes in addition to normal food (18), which is recommended as the preferred nutritional therapy for patients with inadequate dietary intake who can eat with nutritional deficiencies or nutritional risks and without enteral nutritional contraindications by the ESPEN (European Society of Parenteral Enteral Nutrition) guideline (5). In recent years, as the effect of ONS in clinical settings has been confirmed in more and more studies (19, 20), the use of ONS in clinical practice has become more and more extensive. In our longitudinal survey on allo-HSCT patients, about 40% of them accepted ONS in the peritransplantation period. A previous quasi-experimental study on children with acute lymphoblastic leukemia showed that oral nutritional supplements can improve the nutritional status of children, reduce the incidence of complications, and decrease the costs of hospitalization (21). However, there is no report about the role of ONS in allo-HSCT patients recently. Our research revealed that ONS can significantly reduce the risk of consuming <50% of the target energy and protein intake, which indicated that ONS may be of great benefit to help allo-HSCT patients reach their nutritional requirements throughout transplantation. Further experimental studies are needed to verify our recent findings.

Both American and European guidelines emphasized the significance of the assessment of NIS (22, 23). Our analysis revealed distinct temporal patterns: gastrointestinal symptoms (nausea/vomiting/diarrhea) peaked during conditioning (T2) and early post-transplant phases (T3), while mucosal symptoms (oral mucositis, problems swallowing) peaked later (T4), aligning with known cytotoxic effects of conditioning regimens. According to our findings, over half of the allo-HSCT patients experienced at least one NIS during transportation with the prevalence of symptoms varied with distinct transplant periods. This highlights the importance of targeted screening for NIS, known to be related to each stage of transplantation and ensuring appropriate intervention. No appetite, also known as anorexia or loss of appetite, is the gastrointestinal symptom of highest frequency in allo-HSCT patients during transplantation, which has been reported to often occur during preconditioning with a prevalence of 32, 88% on the day of transplantation, and 78% 30 days after transplantation (24). No appetite was also the most common NIS in our survey, but the prevalence was relatively low compared to the previous report, which may be related to the difference in the tools used and the time of the surveys. Loss of appetite occurs in conditions such as psychiatric problems, the use of certain medications, depression, the reduced pleasure of eating due to treatment-related gastrointestinal problems and a variety of alterations in central neurotransmitters (25), which indicates that there is a strong association between loss of appetite and other NIS symptoms. Further research is needed to explore the interactions among nutrition-impacted symptoms in allo-HSCT patients to find effective strategies to reduce the burden of NIS. Importantly, our binary assessment (presence/absence) of NIS precluded severity grading, limiting precise quantification of symptom burden. Future studies should incorporate validated severity scales like the Oral Mucositis Assessment Scale (OMAS) or Common Terminology Criteria for Adverse Events (CTCAE).

Furthermore, our study revealed that patients receiving the FBM conditioning regimen had a significantly higher risk of inadequate oral energy intake (<50% of target) compared to those receiving the FB regimen. This difference may be attributed to the distinct toxicity profiles of the two regimens. The FBM regimen includes melphalan, an alkylating agent associated with higher rates of severe mucositis and gastrointestinal toxicity (e.g., nausea, vomiting, and diarrhea) compared to the FB regimen (fludarabine/busulfan) (7, 26). Melphalan is known to cause profound mucosal damage and prolonged anorexia, which could directly impair oral intake (27). Although our study did not systematically record antiemetic usage between groups, it is plausible that the FBM group required more aggressive antiemetic prophylaxis due to its higher emetogenic potential. However, even with optimal antiemetic support, melphalan-induced mucositis and dysgeusia (“things taste funny”)—which were prevalent in our cohort may limit nutritional intake independently of nausea control (7, 27). Future studies should explicitly compare antiemetic protocols and symptom severity profiles between conditioning regimens to clarify their interactions with oral intake.

### Limitations of study

A limitation of our study is that participants were recruited from a single center, which may lead to biases in patient selection. In addition, a relatively small sample size limits the detection of small differences in the adequacy of intake based on diseased or transplant characteristics. Furthermore, we only recorded a single-day oral intake of the allo-HSCT patients at 7 timepoints, which is merely an indication that such a condition of intake is likely to occur at different periods of peritransplantation, and healthcare providers should further conduct continuous assessments to determine the timing of nutritional support interventions in clinical practice. Due to the lack of a special tool for NIS assessment, only the prevalence of NIS, but not the severity of symptoms, was investigated in the recent study. Moreover, our study focused exclusively on oral intake adequacy and excluded patients requiring parenteral nutrition, which limits the generalizability of findings to the broader allo-HSCT population requiring nutritional support.

## Conclusion

In conclusion, this study provides insight into oral energy and protein intake and extends the small evidence base about the burden of NIS in allo-HSCT patients during the peritransplantation period. With the transplantation, the adequacy of oral energy and protein intake was significantly lower than at admission and returned to the level of admission day on the day before discharge. However, in general, insufficient oral energy and protein intake and the burden of NIS in peritransplantation patients are common, and further attention and intervention on these issues are needed. ONS is a potential approach to increase oral intake, and future studies may further explore the value of ONS in improving nutritional status in allo-HSCT patients.

## Data Availability

The original contributions presented in the study are included in the article/supplementary material, further inquiries can be directed to the corresponding author.
